# Advancing towards Effective Glioma Therapy: MicroRNA Derived from Umbilical Cord Mesenchymal Stem Cells’ Extracellular Vesicles

**DOI:** 10.21315/mjms2019.26.4.2

**Published:** 2019-08-29

**Authors:** Eko Ngadiono, Novi Silvia Hardiany

**Affiliations:** 1 International Class Program, Faculty of Medicines Universitas Indonesia, Jakarta, Indonesia; 2 Department of Biochemistry & Molecular Biology, Faculty of Medicine, Universitas Indonesia, Jakarta, Indonesia

**Keywords:** glioma, umbilical cord, mesenchymal stem cells, extracellular vesicles, microRNA

## Abstract

A glioma, especially a grade IV glioblastoma, is a malignant tumour with a poor prognosis despite growing medical advancements. Researchers have been looking for better and more effective treatments targeting the molecular pathways of gliomas due to glioblastomas’ ability to develop resistance to chemotherapies. Moreover, glioma stem cells (GSC) contribute to maintaining the glioma population, which benefits from its ability to self-renew and differentiate. Recent research has reported that through the introduction of umbilical cord mesenchymal stem cells (UCMSC) into glioma cells, the growth and development of the glioma cells can be downregulated. It has more currently been found out that UCMSC release extracellular vesicles (EVs) containing miRNA that are responsible for this phenomenon. Therefore, this review analyses literature to discuss all possible miRNAs contained within the UCMSC’s EVs and to elaborate on their molecular mechanisms in halting gliomas and GSC growth. This review will also include the challenges and limitations, to account for which more in vivo research is suggested. In conclusion, this review highlights how miRNAs contained within UCMSC’s EVs are able to downregulate multiple prominent pathways in the survival of gliomas.

## Introduction

A glioma is a type of cancer that arises from glial cells. In 2016, the World Health Organization (WHO) updated its terminology in categorising gliomas, which can be categorised according to the tissue from which the cancer arises, including astrocytoma, oligodendroglioma, oligoastrocytoma, and glioblastoma (1). Other than mere histological categorisation, WHO has incorporated the molecular patterns of the tissues (1). Generally, grade I has a low proliferative potential, grade II has an infiltrative nature, and grade IV is the most malignant type (1). Although grade II glioma can be resected surgically, glioma may infiltrate into other brain areas, limiting its complete removal (2). The average survival rate of patients with a grade II tumour is an average of seven years, a rate that has not been improved for the past three decades, based on previous WHO assessment due to a limited number of studies (3). Additionally, some grade II gliomas are able to advance into more malignant types of tumours, worsening the prognosis (3). Unfortunately, having a glioblastoma, a grade IV astrocytoma, is strongly associated with a poor patient outcome and with a median survival rate of approximately six months (4). Despite the advancements of cancer research and treatments, the outcomes, even after these treatments, remain substandard (5). These seemingly tough glioblastomas may arise due to a small number of cancer stem cells (CSC) that help in the development of gliomas (6). This small population of CSC, similar in its role to normal stem cells, allows cancer cells to proliferate as well as to differentiate into various cell phenotypes (7).

In recent years, increasing evidence has shown that the condition media of mesenchymal cells derived from umbilical cords may inhibit the cell cycle and stimulate a senescence effect on gliomas (6). This finding sheds new light on a possible method of treating gliomas, as the umbilical cord mesenchymal stem cells (UCMSC) are able to migrate towards CSC, specifically with high proliferation and low immunogenicity, making them a suitable candidate to fight against CSC (7). However, is still not known which components released by UCMSC exert an inhibitory effect on gliomas. Recently, there have been many reports that have shown microRNAs (miRNAs) possess inhibitory abilities concerning gliomas. Therefore, the inhibitory effects that have been shown by UCMSC may be due to the diverse miRNAs within UCMSC’s extracellular vesicles (EVs), which affect the signal cascade pathway of the glioma (8). Thus, this review will highlight the prominent miRNAs released within UCMSC’s EVs that may limit the survival of gliomas and glioma stem cells (GSC) as potential glioma treatments. Moreover, the molecular mechanisms behind glioma and GSC survival inhibition will be described.

## Normal Functions of UCMSC’s Extracellular Vesicles and MicroRNAs

The discovery of miRNA has changed the perspective of researchers in the regulation of mRNA. Moreover, miRNAs were once considered to be inoperable, noncoding RNA. MiRNAs are single-stranded RNA with a length of approximately ~22 nucleotides (9). RNA polymerase II within the nucleus helps in the formation of a unique stem-loop structure, called pri-miRNA, through transcription (10). The structure of pri-miRNA is similar to general mRNA, as it is equipped with a 5′ cap and a poly-A-tail (11); additionally, and it is cleaved by the RNAse III endonuclease attached to the microprocessor (DGCR8/DROSHA) complex in the nucleus, leaving only around 65–70 nucleotide structures, called precursor-miRNAs (pre-miRNAs) (11). This processing of pre-miRNAs by the microprocessor is closely regulated by the Smad (11). The resulting pre-miRNAs are transported out of the nucleus through the nuclear exporting factor 5 (EXP 5) (12). Once pre-miRNAs arrive in the cytoplasm, the RNAse III endonuclease component of another protein, called Dicer, working together with TARBP2 and PRKRA, cleaves pre-miRNAs into miRNAs (13). The resulting double-stranded miRNAs are separated into two: a guide strand, which will attach to RNA-induced silencing complexes (RISC), and a passenger strand, which will be discarded (10). RISC-carrying, mature miRNAs will detect 3′ untranslated regions (UTR) of their complementary strands and repress the target mRNA translation, the mRNA degradation, or both (9). Similar to other nuclear regulation, the expressions of miRNAs are also affected by epigenetic aspects, such as DNA methylation as well as histone modifications (10). In general, miRNAs work through post-transcription control, limiting the mRNAs to be translated.

UCMSC secrete multiple cytokines as well as EVs to communicate with neighbouring cells in a paracrine manner (14, 15). Cytokines are proteins that are freely secreted to the extracellular environment and that bind to a cell’s receptor. On the other hand, EVs serve as a vehicle that protects vulnerable compounds, such as RNA or protein, from the extracellular environment. The released EVs from the donor cells can be taken by recipient cells through endocytosis, delivering the contained materials into the recipient cell’s cytosol or intracellular organelles (9). Interestingly, EVs secreted by UCMSC contain miRNAs, for which the complementary bases are located within gliomas, negating RNAs’ potent effect for gliomas’ survival (16, 17). Recently, a range of miRNAs within UCMSC’s EVs were discovered (14). However, the full mechanism of how EVs are endocytosed into gliomas is yet unknown. Furthermore, the mechanism of the released miRNAs that search for their complementary mRNAs within gliomas is also unknown. Nevertheless, evidence has shown that EVs from UCMSC were successfully internalised by gliomas through a distinct mechanism, inhibiting proliferation and stimulating gliomas’ apoptosis in vitro (18). Multiple types of miRNAs (as shown in Table 1) and their proposed mechanisms within gliomas will be described below.

### MicroRNAs, Found within UCMSCs’ EVs, Downregulate Essential Gliomas and Glioma Stem Cells’ Molecular Pathways

#### MiR-199a-3p limits glioblastoma survival through the AKT-mTOR pathway, leading to Survivin’s downregulation

Though UCMSC’s EVs do not contain miR-199a-3p specifically, they contain its precursor, miR-199a (17). It has been shown that glioblastomas, introduced with miR-199a-3p, reveal low overall proliferation and low expression of proteins in the AKT-mTOR pathway, including mTOR, AKT and P70S6K (16). The downregulation of protein in this AKT-mTOR pathway initiates the glioma autophagy mechanism (19). Furthermore, AKT-mTOR downstream signalling will eventually lead to a potent protein called Survivin (20).

Survivin is present in highly divisive cells, such as developing embryos and, unfortunately, cancer (21). However, it is less likely to locate Survivin in terminally differentiated cells (21). The increased expression of Survivin worsens the outcome of gliomas (22). Addressing Survivin as potential therapeutic target slows the progression of gliomas by inhibiting invasion and reducing their proliferation rate (23). Moreover, suppressing Survivin is correlated with decreased resistance to chemotherapy and radiation therapy (24, 25). Survivin is critical in affecting gliomas’ survival due to its dual functions, that is, cell division and its effect on regulating caspase 9 (the internal apoptosis pathway) (26, 27). It can be said that damage to Survivin’s ability in a normal cell will lead normal cells into malignancies in agreement with the two hallmarks of cancer, resisting apoptosis and the cell’s ability to proliferate indefinitely (28). Therefore, the effort in blocking the upstream pathway of Survivin, the AKT-mTOR pathway, is crucial in limiting the aggressiveness of gliomas.

#### MiR-410 inhibition of GSC and the survival of glioblastomas through MET

MET is a hepatocyte growth receptor (HGR), and upon binding of the hepatocyte growth factor, phosphorylates AKT to prevent cell apoptosis through the inhibition of caspase (29). It was further shown that MET regulates the signalling system of GSC by increasing their proliferation, migration rate, and almost all critical survival ability (30). UCMSC contain miR-410 within their EVs (31). The various outcomes in transferring miR-410 have been in conflict. Moreover, the over-expression of miR-410 has been shown to increase the survival of lung adenocarcinoma, liver and colorectal cancer (31, 32). In contrast, the introduction of miR-410 into a glioblastoma results in suppressing the growth of the glioblastoma by inhibiting the expression of MET (33). Moreover, the downregulation of MET sensitises the GSC to ionising radiation (29). Thus, the downregulation of MET may be a prominent solution, as it suppresses the aggressiveness of the GSC and glioblastomas in any aspects.

#### MiR-146a inhibition of the growth of gliomas and GSC through Notch1 signalling

An analysis was performed by Ti et al. to estimate the miRNAs in the EVs of UCMSC, comparing them to the EVs of human fibroblasts (34). It showed that three miRNAs were upregulated where only two miRNAs were described, miR-146a and miR-181 (34). The exposure of miR-146a prevents malignant astrocytes from being turned into GSC (35). It has been shown that miR-146a targets the expression of the Notch1 protein, which is often over-expressed in gliomas and glioblastomas, by downregulating its transcription pathway and, consequently, reducing the epidermal growth factor receptor (EGFR) downstream pathway activities (35, 36). EGFR is often amplified and over-expressed in cancer cells and, unexceptionally, in gliomas (37). Moreover, around half of the over-expressed EGFR are found to express the mutant EGFRvIII variant, resulting in the failure of ligand binding and autophosphorylation (38, 39). Neither the expression of EGFRvIII nor the over-expression of EGFR decreases the aggressiveness of gliomas (40, 41). One of the reasons for this may be that the constitutive activation of EGFRvIII and over-expressed EGFR in gliomas activates the downstream pathways that play an essential role in promoting the survival of gliomas through STAT3, MAPK and AKT (42). AKT activation is more prominent in both cases while STAT3 and MAPK activation varies (42).

As aforementioned, activated AKT shares a part in guiding the multiple pathways responsible for the malignant behaviour of gliomas. MiR-146a generates an anti-glioma effect by preventing AKT from becoming phosphorylated (36). In addition, evidence has shown that miR-146a is able to suppress other EGFR downstream pathways, including PI3K, K-Ras, cyclin D1 and MMP9 (35). PI3K is an upstream component of a complex network responsible for activating AKT as well as mTOR (43). Eventually, the activation of PI3K will promote growth and inhibit apoptosis in gliomas (43). Activated k-Ras will eventually lead to PI3K/AKT, NF-kB and ERK signalling pathways (44). Similarly, its aberrant activation is also associated with carcinogenesis (44). Part of Cyclin D1’s function is regulating the cell cycle. Downregulating Cyclin D1 has been shown to be effective in halting cell proliferation and promoting apoptosis when cisplatin was administered (45). Lastly, MMP9 is a component of the matrix metalloproteinases (MMP) family, which is responsible in degrading the extracellular matrix (46). MMP9 has been speculated to be responsible for the invasion of gliomas; furthermore, high expression of MMP9 is correlated with high cell growth and has been shown to be highly present in more aggressive types of gliomas (46).

#### Let-7b suppression of GSC and glioblastomas through the regulation of cell cycle components and apoptosis

Previously, UCMSC treated with lipopolysaccharide were found to contain Let-7b in their EVs (47). Let-7b is responsible for halting the growth and migration of glioblastomas and GSC by directly targeting the 3′ UTR region of E2F2, a transcription factor responsible in the cell cycle and tumorigenesis (48). The over-expression of Let-7b also suppresses cyclin D1 protein expression in glioblastomas (45). Lastly, Let-7b downregulates a distinct component called the inhibitor of the nuclear factor kappa-B kinase subunit epsilon (IKBKE) in glioblastomas (49). IKBKE is often found to be over-expressed in gliomas and is believed to play a role in inhibiting apoptosis through the NF-kB pathway (50). Furthermore, IKBKE activates NF-kB by phosphorylating its inhibitor, the inhibitor kappa B (50). The NF-kB pathway is very complex and is responsible for various cell controls (51). NF-kB downregulation has also been associated with suppressed vascular endothelial growth factor (VEGF) and IL-8, a component and cytokine, respectively, responsible for angiogenesis (52). Therefore, Let-7b is potent in downregulating NF-kB through IKBKE. Nevertheless, research has shown that IKBKE and Let-7b are components of the regulatory feedback loop in which a decrease of one component will increase the other (53). Thus, instead of downregulation, research has suggested a block of the circuit as a method for glioblastoma treatment (53).

#### MiR-181 family targeting of multiple cellular pathways in glioblastomas and GSC

As previously mentioned, mir-181 was upregulated within UCMSC’s EVs (34). However, it was not mentioned which miR-181 members were upregulated. Therefore, each of the members will be discussed herein as each member presents potential as a glioma treatment. The miR-181 family contains four members, including miR-181a, miR-181b, miR-181c and miR-181d (54). In general, miR-181 oligonucleotide targets cyclin B1 3′ UTR and downregulates its expression in gliomas and glioblastomas (55). Cyclin B1 is important for cell cycle progression and is often dysregulated in gliomas (56).

MiR-181a targets the 3′ UTR region of Notch2 in glioblastomas (57). The downregulation of Notch2 was reported to exert the same outcome as the downregulation of Notch1 (58). Notch2 was also suspected of participation in the EGFR/P13K/AKT pathway in conjunction with Notch1 (58). However, the exact mechanism of Notch-2 in gliomas is still debatable (57). The over-expression of Notch2 stimulates the malignancy of gliomas and GSC while the introduction of mir-181a successfully inhibits them (57). Another study confirmed miR-181a as a potential glioblastoma treatment by sensitising the glioblastomas to radiation through B-cell lymphoma 2 protein (Bcl-2) downregulation, an anti-apoptotic protein (59, 60).

MiR-181b sensitises gliomas and GSC to various chemotherapies, such as temozolomide and teniposide, by targeting the 3′ UTRs of both MDM2 and MEK1, respectively (61–63). MEK1 is one component in the MAPK pathway, a pathway that is often highly expressed in gliomas responsible for regulating cell apoptosis, proliferation, and resistance to chemotherapy (61). Often over-expressed in glioblastomas (64), MDM2 is an E3 ubiquitin ligase that degrades a tumour’s suppressor protein, called p53 (64). MiR-181b suppresses phosphorylated MDM2, reverting p53 degradation and sensitising gliomas to teniposide (62). Other research has discovered that miR-181b downregulates KPNA4 in glioblastomas, a protein within an elaborate pathway that is responsible for activating NF-kB (65). As mentioned previously, aberrant NF-kB is often correlated with the proliferation of gliomas, in which its expression is best kept in control. MiR-181b was also shown to suppress glioblastoma growth in vivo (65).

MiR-181c is highly responsible in controlling the invasion and proliferation of gliomas. Similar to miR-181a, miR-181c has been suspected of binding at Notch2 UTRs, downregulating its expression (66). MiR-181c inhibits the epithelial-mesenchymal transition (EMT) of glioblastomas through the downregulation of N-cadherin and Vimentin while E-cadherin is upregulated (67). EMT is a term that refers to when epithelial cells lose their adhesion and migrate out as mesenchymal cells (68). It is considered a factor for glioma invasiveness (68). Furthermore, the involvement of E/N-cadherin is closely related to the EMT of gliomas. E-cadherin is rarely expressed in gliomas, whereas N-cadherin expression is often upregulated (68). Meanwhile, the over-expression of Vimentin has resulted in the increased growth of gliomas, as it is believed to act as a chaperone for various protein kinases (69). It has been further concluded that TGF-β plays an essential role in controlling glioma invasion, in which miR-181c is able to inhibit its downstream pathway (67). TGF-β is a cytokine of which the downstream pathway is associated with proliferation, angiogenesis, invasion, and immunosuppression (70). Even worse, gliomas actively secrete TGF-β, self-facilitating its growth (70). Therefore, blocking TGF-β cytokine activity poses a great advantage for the treatment advancement of gliomas.

MiR-181d has been reported to bind directly to methyl-guanine-methyl-transferase (MGMT) 3′ UTR, sensitising glioblastomas to temozolomide (71). MGMT is a protein responsible for repairing TMA-induced DNA damage that leads to temozolomide therapy failure (71). Furthermore, miR-181d suppresses multiple signalling activities, which include MAPK/ERK and P13K/AKT pathways through K-ras activation (44). MiR-181d directly targets the 3′ UTR of K-ras and Bcl-2 (44). As previously discussed, these pathways and components are essential for glioma tumourigenesis. Moreover, miR-181d even successfully inhibits the growth of glioblastomas in vivo (44).

#### MiR-145 limitation of GSC and GBM aggressiveness by ABCG2 and ADAM19, respectively

MiR-145 has been found to be contained in UCMSC’s EV’s (17), and it serves as a potent regulator in downregulating the ATP-binding cassette, sub-family G, member 2 (ABCG2) expressions in GSC (72). It is widely known that ABCG2 is a transporter protein that transports xenobiotic drugs out from tumour cells. An important factor, GSC is resistant to multiple chemotherapeutic drugs (73). Another study reported that by downregulating ABCG2, GSC are more vulnerable to temozolomide (74). Moreover, ABCG2 serves as one factor of gliomas’ stemness and roles in invasion as well as migration (75, 76). MiR-145 directly targets ADAM19 3′ UTR downregulating a glioblastoma’s ability for EMT (77). The resulting expression of N-cadherin, E-cadherin, and Vimentin show similar results when miR-181c is introduced (77).

#### Multiple miRNAs obtained in UCMSC EVs potentiate gliomas’ survival

Gliomas are known to express multiple kinds of cytokines to enhance their survival and migration. However, other than expressing cytokines benefiting their own growth, it was also discovered that gliomas release miRNAs, found in the serum of patients’ blood (78). MiRNAs released by gliomas may affect important regulations in many biological processes. Therefore, a full range of miRNAs were addressed, and their expressions were calculated. Several miRNAs, such as miR-1252, miR-4434, and miR-4669, have been found to be upregulated significantly (78). Interestingly, these miRNAs are also found within UCMSC’s EVs in their primary form (14). However, less is known concerning their function in the development of gliomas’ survival, let alone their molecular mechanisms. It is therefore suggested to conduct more research on these miRNAs as they pose a potential treatment or threat to gliomas.

## Perspectives and Challenges

MiRNAs contained within UCMSC’s EVs pose a promising prospect as a treatment for gliomas in the future. The UCMSC’s EVs are equipped with diverse miRNAs that inhibit multiple downstream pathways accountable for gliomas as well as GSC survival. These miRNAs regulate cell signalling that is important for proliferation, angiogenesis, invasion, chemotherapy effectiveness, and apoptosis. Furthermore, prominent signalling components and pathways that are often over-expressed in gliomas, such as MAPK/ERK, P13K/AKT, MAPK and cell cycle regulators, were downregulated. Each miRNA possesses capabilities in inhibiting gliomas through various pathways that will intersect eventually and amplify the inhibition effects of miRNAs. In addition to inhibiting prominent pathways, some miRNAs inhibit the same components of gliomas’ aggressiveness, including Notch2, Bcl-2, K-ras and the AKT protein. In addition, an miRNA is not only released by UCMSC within the extracellular vesicle, but a range of miRNAs are also released by certain types of cancer (12). Certain cancers release unique profiles of miRNAs that differ from one cancer cell to another (12). Therefore, miRNAs can not only be used for treatment purposes by suppressing over-reactive cancer cells’ mRNAs, but they can also be used as cancer biomarkers for diagnostic purposes (12).

Nevertheless, there are still challenges needing to be solved in using UCMSC as a future treatment for gliomas. Cytokines released by UCMSC function as growth factor for gliomas instead of restricting them, for example, IL-6, GRO, MCP-1 and IL-8 (7). IL-6 activates the JAK/STAT3 pathway in human gliomas, an activation that is correlated with the proliferation and invasion of gliomas (79). However, the JAK/STAT3 pathway is not regulated through any identified miRNAs derived from UCMSC’s EVs. GBM exerts the IL-8 autocrine mechanism and expresses a high number of IL-8 receptors, such as CXCR1 and CXCR2 (80). Therefore, IL-8 is suspected to be a potent regulator in glioma survival. Moreover, it was reported that IL-8 plays a certain part in regulating NF-kB in gliomas (80). In this case, though miRNAs inhibit NF-kB through the downregulation of IKBKE, the downstream mechanism of IL-8 should be taken into consideration. Meanwhile, the detailed molecular mechanism involving MCP-1 and GRO in gliomas is still unclear.

More research still needs to be conducted considering many factors that may affect miRNAs’ efficacy derived from UCMSC’s EVs as a treatment for gliomas. The research reviewed in this article was performed only by introducing a type of miRNA. None of the research performed introduced multiple miRNAs to measure the outcome. Therefore, it is suggested to perform more research in the future to understand the inter-communication between multiple miRNAs in gliomas, reflecting the actual settings of UCMSC’s EVs’ delivery. Furthermore, in vivo research and clinical studies are suggested. Also, the outcome preceding miRNAs’ delivery might differ depending on which glioma cell lines are used. Last but not least, the majority of glioma tissues used for miRNAs quantification are collected and performed in mainland China. Therefore, it is suggested that researchers planning to perform similar research take genetic bias into consideration.

## Conclusion

This review summarises a range of potential miRNAs as glioma treatments contained within UCMSC’s EVs. Additionally, this review highlights the utilisation of these miRNAs in inhibiting multiple prominent signalling pathways responsible for overall glioma and GSCs’ survival, proposing this method as an effective glioma treatment in the future.

## Figures and Tables

**Table 1:** Inhibitory effect of microRNA derived from UCMSC’s EVs against glioma

Potential miRNA	Downregulated pathway	Downregulated components	Potential cells	Potential outcome	Reference
miR-199a-3p	Akt-mTOR pathway	mTOR, Akt, and P70S6K	Glioblastoma	Reduced proliferation, autophagy	(16, 17, 19)
miR-410	-	MET	Glioblastoma, GSC	Sensitising to ionising radiation	(29, 30, 33)
miR-146a	EGFR signalling pathway	Notch1 protein, P13K, K-Ras, Cyclin D1, and MMP9 inhibiting Akt phosphorylation	Glioma, GSC	Decreased in aggressiveness	(34, 35)
Let-7b	NF-kB	E2F2, Cyclin D1, IKBKE	Glioblastoma, GSC	Reduced growth and migration	(45, 47, 48, 49)
miR-181 family	Cell cycle	CyclinB1	Glioma, Glioblastoma	Reduced proliferation, autophagy	(34, 55)
miR-181a	EGFR/P13K/Akt	Notch 2, Bcl-2	Glioblastoma, GSC	Sensitising to ionising radiation	(57, 59)
miR-181b	MAPK, NF-kB	MEK1, MDM2, KPNA4	GSC, Glioblastoma	Sensitising to temozolomide and teniposide	(61, 62, 63, 65)
miR-181c	TGF-β downstream pathways	Notch2, N-cadherin, Vimentin Upregulated E-cadherin	Glioblastoma	Decreased in invasiveness	(66, 67)
miR-181d	MAPK/ERK and P13K/AKT	MGMT, K-ras, Bcl-2	Glioblastoma	Decreased glioblastoma growth *in vivo*, sensitising to temozolomide	(44, 71)
miR-145	-	ABCG2, ADAM19	GSC, glioblastoma	Sensitising to temozolomide, reduced invasion	(17, 72, 77)
